# An Active Site Aromatic Triad in *Escherichia coli* DNA Pol IV Coordinates Cell Survival and Mutagenesis in Different DNA Damaging Agents

**DOI:** 10.1371/journal.pone.0019944

**Published:** 2011-05-17

**Authors:** Ryan W. Benson, Matthew D. Norton, Ida Lin, William S. Du Comb, Veronica G. Godoy

**Affiliations:** Department of Biology, Northeastern University, Boston, Massachusetts, United States of America; University of Massachusetts Medical School, United States of America

## Abstract

DinB (DNA Pol IV) is a translesion (TLS) DNA polymerase, which inserts a nucleotide opposite an otherwise replication-stalling *N^2^*-dG lesion *in vitro*, and confers resistance to nitrofurazone (NFZ), a compound that forms these lesions *in vivo*. DinB is also known to be part of the cellular response to alkylation DNA damage. Yet it is not known if DinB active site residues, in addition to aminoacids involved in DNA synthesis, are critical in alkylation lesion bypass. It is also unclear which active site aminoacids, if any, might modulate DinB's bypass fidelity of distinct lesions. Here we report that along with the classical catalytic residues, an active site “aromatic triad”, namely residues F12, F13, and Y79, is critical for cell survival in the presence of the alkylating agent methyl methanesulfonate (MMS). Strains expressing *dinB* alleles with single point mutations in the aromatic triad survive poorly in MMS. Remarkably, these strains show fewer MMS- than NFZ-induced mutants, suggesting that the aromatic triad, in addition to its role in TLS, modulates DinB's accuracy in bypassing distinct lesions. The high bypass fidelity of prevalent alkylation lesions is evident even when the DinB active site performs error-prone NFZ-induced lesion bypass. The analyses carried out with the active site aromatic triad suggest that the DinB active site residues are poised to proficiently bypass distinctive DNA lesions, yet they are also malleable so that the accuracy of the bypass is lesion-dependent.

## Introduction

Replicative DNA polymerases are multi-protein complexes responsible for synthesizing a high fidelity copy of a cell's genome. Persistent lesions on the template DNA, which DNA repair pathways have failed to recognize, result in stalling of DNA replication, a potentially lethal event [1]. To avoid lethality, specialized DNA polymerases insert deoxynucleotides (dNTPs) opposite replication-blocking DNA lesions in a process known as translesion synthesis (TLS). This is largely a low fidelity process usually resulting in elevated mutagenesis [1], [2]. In *Escherichia coli* there are three TLS polymerases that are regulated by the SOS gene network, one of the cellular responses to DNA damage and environmental stress [1], [3]. The *polB* gene encodes the B family DNA Pol II, while the *dinB* gene and the *umuDC* operon encode the two Y family DNA polymerases, DNA Pol IV and DNA Pol V respectively [1], [4], [5], [6], [7]. DinB is of particular interest because of its evolutionary conservation [1], [6], [8] and its high basal intracellular concentration (∼250 nM) [1], [9], [10]. Indeed, this is approximately 17 fold higher [10] than that of DNA Pol III complex (the replicative DNA polymerase, 15 nM; [9]) and is similar to that of the processivity clamp (β-clamp, 250 nM; [11], [12]), an essential replication factor known to both recruit all DNA polymerases to the replication fork and manage their activity in the cell [13], [14].

*E.coli* cells lacking the *dinB* gene (Δ*dinB*) are sensitive to nitrofurazone (NFZ) and 4-nitroquinoline-1-oxide (4-NQO) [15], [16], reagents that create persistent DNA lesions on the *N^2^* group of deoxyguanosine (*N^2^*-dG) [17], [18]. Recent evidence suggests that DinB and its homologues can also perform TLS of lesions that are the product of alkylation of DNA bases [19], [20], [21]. Alkylating agents are both a byproduct of the cell's metabolism and also come from diverse exogenous sources generating DNA damage in prokaryotic and eukaryotic cells [1], [22], [23], [24], [25]. In addition, alkylating agents are used as anti-cancer chemotherapeutic agents, [1], [26], [27], underscoring the significance of understanding the cellular mechanisms of alkylation lesion tolerance.

It is known that base excision repair pathways are the primary cellular response to alkylation damage [1], [28], [29], [30], [31], [32], though Y family DNA polymerases are also part of this response [1], [19], [20], [21]. These polymerases likely bypass 3-methyladenine (3-meA; [1], [19], [20], [21], [33], [34]), a prevalent alkylation lesion that persists on the DNA and brings about replication fork stalling and cell death [20], [21], [35], [36]. Indeed, *E. coli* strains lacking the *dinB* gene (*ΔdinB*) are sensitive to several alkylating agents, such as methyl methanesulfonate (MMS; [19]). Similar sensitivity is found in eukaryotic cells deficient in TLS polymerases [20], [21]. Thus, the evidence so far indicates that if DNA repair pathways do not effectively recognize 3-meA, Y family DNA polymerases are critical in the cell's response to alkylation damage [19], [20], [21]. Unfortunately, 3-meA has a very short *in vitro* half-life [21], [37], making difficult to directly investigate the bypass mechanisms of this alkylation lesion.

Most of our knowledge in regard to the active site of DinB has been acquired through studies with reagents that generate *N^2^*-dG lesions [15], [16]. However, it is not known which aminoacids in the DinB active site are important for the bypass of alkylation lesions, e.g. most likely 3-meA. It is also unclear whether the same active site residues are involved in the bypass or its fidelity of both alkylation and *N^2^*-dG lesions. Structural modeling predicts that Pol κ (the mammalian DinB homologue) could accommodate either the *N^2^*-dG or 3-meA minor groove adducts in its active site in a conformation that would allow both insertion and extension from either adduct [20].

**Figure 1:**
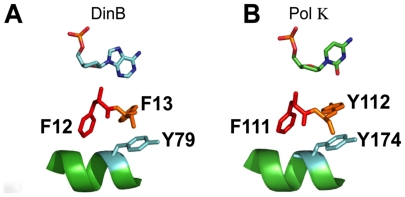
Homologous *E. coli* DinB and human Pol κ aromatic triads appear similarly positioned in the active site. The near identical conformation of the aromatic triads of (**A**) DinB (Pol IV) (F12, F13 and Y79) and (**B**) Pol κ (F111, Y112, and Y174) suggests these residues could be required for TLS activity of Pol κ. The DinB structure is from an *in silico* model generated in collaboration with A. Abyzov and V. Ilyin [40]. Image generated using PyMOL (DeLano, W.L. The PyMOL Molecular Graphics System (2002) DeLano Scientific, San Carlos, CA, USA.). Pol κ structure was rendered using the pdb 3IN5 with PyMOL.

We studied a triad of aromatic residues (Fig. 1) that is conserved in Y family DNA polymerases including Pol η and Pol κ [38], [39] and used as a control the strictly catalytic aspartic acid 103 (D103). This is known to be critical for DNA synthesis and thus unable to complement a Δ*dinB* strain [16], [40], [41]. Here, we describe the analysis of the aromatic triad residues of DinB in response to DNA damage generated by treatment with MMS or NFZ, reagents that create respectively alkylation or *N^2^*-dG lesions *in vivo*. This report describes the effect of changing the aromatic triad residues to those of different polarity or size on both survival and DNA damage-induced mutagenesis. This type of analysis has permitted us to learn about the intricacies of *in vivo* DinB lesion bypass activities. We infer that the classical catalytic and the highly conserved DinB active site “aromatic triad” are necessary for TLS of alkylation lesions. Remarkably, the aromatic triad also serves the function of governing *in vivo* TLS fidelity, which seems to be lesion-dependent.

## Results

### DinB active site residues are important for survival in MMS

The two catalytic activities of DinB, phosphodiester bond formation (i.e. DNA synthesis) and lesion bypass are separable [16]. Each activity can be tested *in vivo* by measuring survival of cells lacking the chromosomal copy of the *dinB* gene (Δ*dinB*) after treatment with a DNA damaging agent. Because some alkylation lesions are chemically unstable (e.g. 3-meA), we took advantage of this genetic approach to determine whether Δ*dinB* cells expressing plasmid-borne *dinB* alleles with mutations in the aromatic triad could survive MMS treatment.

Low copy number plasmids expressing different *dinB* alleles from the native SOS inducible promoter were introduced into Δ*din*B by transformation (Table S1). Cells were then assayed for survival at various concentrations of MMS. As expected, the plasmid carrying native *dinB^+^* rescues Δ*dinB* treated with MMS (Figs. S1 and 2), while the strain expressing the catalytically inactive *dinB(D103N)* variant [41] is highly sensitive to MMS treatment (Figs. S1 and 2). Unexpectedly, Δ*dinB* cells expressing *dinB(F13V)* did not show the prototypical highly NFZ sensitive phenotype [16] upon treatment with MMS (Figs. S1 and 2).

**Figure 2:**
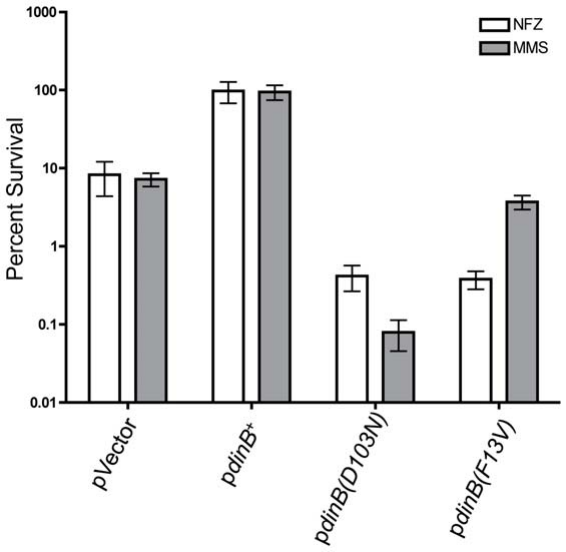
Δ*dinB* is rescued from MMS and NFZ lethality only by *dinB* ^+^ (**A**) Neither plasmid-borne DinB(D103N) nor DinB(F13V) rescue Δ*dinB* cells treated with MMS (7.5 mM) or NFZ (0.008 mM). Enhanced sensitivity to MMS is observed in Δ*dinB* with DinB(D103N) (gray bars). Δ*dinB* strains with either DinB(F13V) or DinB(D103N) variants exhibit an enhanced cellular sensitivity to NFZ compared to Δ*dinB* (pVector, white bars) as previously reported [16]. Error bars represent the standard deviation of the mean from at least 3 independent experiments.

**Figure 3:**
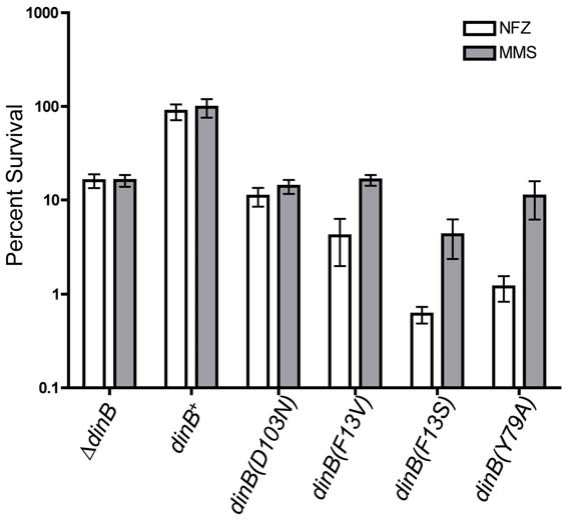
MMS or NFZ survival phenotypes of strains with catalytic or TLS deficient *dinB* chromosomal alleles. Cells carrying a single chromosomal copy of the catalytic deficient *dinB(D103N)*, the TLS deficient *dinB(F13V)*, *and* other *dinB* alleles were assayed for survival as indicated in material and methods with MMS (7.5 mM) or NFZ (0.008 mM). Error bars represent the standard deviation of the mean from at least 3 independent experiments.

We were intrigued by the enhanced cellular sensitivity to MMS (Δ*dinB* with the *dinB(D103N)*, gray bars; Fig. 2) or NFZ (Δ*dinB* with either the *dinB(D103N)* or *dinB(F13V)*, white bars; Fig. 2) compared to Δ*dinB*. The simplest explanation is that the phenotype is due to high DinB intracellular concentrations, despite being expressed under the SOS-regulated native promoter and from low copy number plasmids. Increased intracellular concentrations of DinB variants may somehow have a more deleterious effect on survival than lack of DinB. Thus, the *dinB(D103N)* and *dinB(F13V)* alleles (Table S1) were crossed onto the chromosome replacing *dinB* ^+^, as indicated in Materials and Methods. Consistent with the hypothesis, we find that cells with a chromosomal copy of *dinB(D103N)* are no longer highly sensitive to MMS (compare ∼100 fold more killing than Δ*dinB* in Fig. 2 with the same lethality as Δ*dinB* in Fig. 3) or NFZ at any of the concentrations tested, and survive treatment as ***Δ*** *dinB* cells (Fig. 3). Conversely, cells with *dinB(F13V)* in the chromosome remain more sensitive to NFZ than Δ*dinB*, though the extent of sensitivity is less dramatic in the chromosome (∼10 fold, white bars; Fig. 3). These data demonstrate that the observed exacerbated sensitivity phenotypes of Δ*dinB* strains with plasmid-borne TLS deficient *dinB* alleles are not solely due to elevated intracellular concentrations of these proteins. However, the mechanism(s) underlying this phenomenon is/are not fully understood.

### The aromatic triad is required for *in vivo* DinB TLS

**Figure 4:**
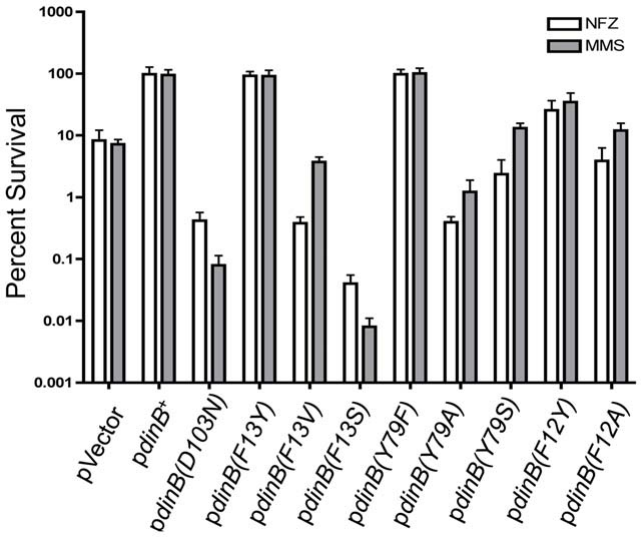
An aromatic triad in the DinB active site is required for Δ*dinB* survival upon treatment with MMS or NFZ. Δ*dinB* harboring the *dinB* alleles with mutations at position 12, 13, and 79 were treated with MMS (7.5 mM; gray bars) or NFZ (0.008 mM; white bars). Treatments were carried out as described in materials and methods. Error bars represent the standard deviation of the mean from at least 3 independent experiments. Only top error bars are shown for clarity.

In an effort to gain insights into the TLS activity of DinB in alkylation lesion bypass, we looked for conserved residues in the DinB active site that could be as important as F13 in DinB *N^2^*-dG TLS. This analysis, carried out with a large number of DinB sequences (>100) in both prokaryotic and eukaryotic organisms (including the DinB human homologue Pol κ), surprisingly shows that phenylalanine 13 (F13) is only somewhat conserved (42%). However, if the analysis also considers tyrosine, a structurally similar residue, then F13/Y13 becomes 97% conserved. We hypothesized that the aromatic ring of tyrosine or phenylalanine fulfills an identical role in lesion bypass. We constructed a DinB derivative with a tyrosine at position 13 instead of phenylalanine (p*dinB(F13Y)*) in the same low copy number plasmid mentioned in the above section, and introduced it by transformation into Δ*dinB*. As predicted, the Δ*dinB*/p*dinB(F13Y)* strain has the same level survival after MMS or NFZ treatment as cells with wild-type DinB^+^ (Fig. 4).

We have shown (Figs. S1, 2 and 3) that F13 is important for the TLS of alkylation lesions, but strains expressing this allele survive better in MMS than in NFZ regardless of the allele location (Figs. 2 and 3). F13 was also changed to alanine or serine. Unlike *dinB(F13V)*, both *dinB(F13A)* (data not shown) and *dinB(F13S)* (Fig. 4) result in decreased survival of Δ*dinB* cells upon NFZ or MMS treatment independent of whether the *dinB* alleles are expressed from a plasmid (Fig. 4) or from the chromosome (Fig. 3). It can be inferred from these data that the role of the F13 residue in the DinB bypass of alkylation DNA lesions is likely different from its role in *N^2^*-dG bypass, but is nevertheless essential for the bypass of MMS-derived lesions *in vivo*.

The two other aromatic residues that are also highly conserved (>95% conservation among DinB sequences) and happen to be in close proximity to F13 in the DinB tertiary structure are Y79 and F12 (Fig. 1). The conservation is true even at the structural level (Fig. 1, compare DinB and Pol κ) suggesting that, unlike F13, both the aromatic ring and the polarity of these residues might be equally important for lesion bypass.

We changed each one of these residues, and assessed their function by measuring survival of Δ*dinB* carrying the various *dinB* alleles upon MMS or NFZ treatment. Y79 or F12 were changed to the aromatic residue with the opposite polarity (phenylalanine or tyrosine respectively) or to the non-aromatic residues alanine or serine.

There is no measureable survival defect for the *ΔdinB* strain upon MMS treatment when the conserved Y79 residue is replaced by phenylalanine (Fig. 4). However, Δ*dinB* cells expressing *dinB(Y79A)* show an enhanced sensitivity to MMS or NFZ compared to Δ*dinB* (pVector; Fig. 4). A similar enhanced sensitivity, though not to the same degree, was observed in NFZ-treated cells when the *dinB(Y79A)* allele is expressed from the chromosome (Fig. 3). In contrast, the result of exchanging the tyrosine for a non-aromatic amino acid of the same polarity, serine, leads to poor survival in MMS or NFZ treatment, similar to that shown by the *ΔdinB* strain (Fig. 4).

*ΔdinB* expressing the plasmid-borne *dinB(F12Y)* allele, however, show reduced survival in MMS or NFZ when compared to cells carrying p*dinB^+^*, but survive better than *ΔdinB*, suggesting that TLS is lessened but not abolished in this variant (Fig. 4). This is confirmed by changing F12 to alanine (Fig. 4), which results in survival similar to that of cells lacking *dinB*. We also investigated the effect of changing F12 to a non-aromatic residue of the opposite polarity and found that the F12S mutation does abolish TLS activity *in vivo*, similar to the F12A mutation (data not shown).

Taken together, the data demonstrate that the aromatic triad consisting of F12, F13, and Y79 are all needed for survival in MMS. The relevance of each residue in TLS varies depending on the lesion and is independent of the location (plasmid or chromosome) of the *dinB* TLS deficient allele. Importantly, the absolute requirement of these residues for DinB lesion bypass, and the evolutionary conservation of these aromatic residues, suggest the importance of corresponding residues in DinB homologues, such as human Pol κ (Fig. 1).

### Survival effects of various *dinB* alleles expressed in *ΔdinB* depend on both the lesion and the DinB processivity clamp-binding motif

**Figure 5:**
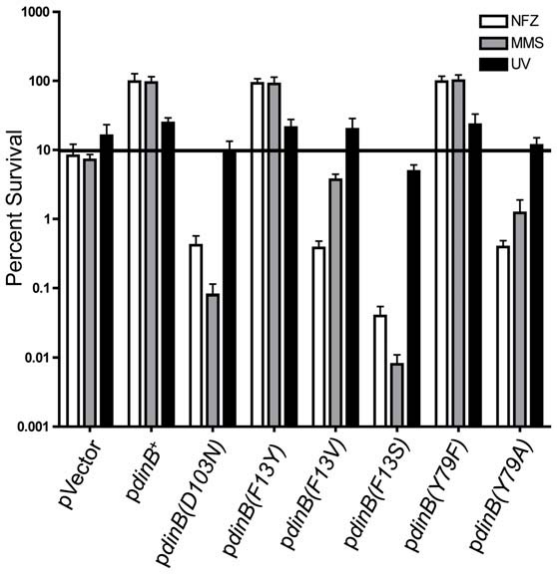
*dinB* deficient alleles affect Δ*dinB* survival only upon treatment with reagents that generate DinB cognate lesions. Δ*dinB* strains carrying the plasmid-borne variants of DinB were compared at levels of UV (37 J/m^2^) at which Δ*dinB* and *dinB* ^+^ are equally killed (black bars). Significant variations in survival were only observed upon comparable levels of MMS (7.5 mM; gray bars) or NFZ (0.008 mM; white bars) treatments. Error bars represent the standard deviation of the mean from at least 3 independent experiments. Only top error bars are shown for clarity.

We asked whether the survival phenotypes of *ΔdinB* expressing various DinB variants were independent of the DNA damaging agent used to treat cells. We took advantage of the enhanced sensitivity phenotype observed in *ΔdinB* strains such as those carrying plasmid-borne *dinB(D103N), dinB(F13S), and dinB(Y79A)* (to MMS or NFZ), or *dinB(F13V)* (to NFZ). We have already shown (Fig. 3) that the increased sensitivity of the *ΔdinB*/p*dinB(D103N)* strain to MMS or NFZ compared to ***Δ*** *dinB*, is likely due to elevated intracellular concentrations. Nonetheless, when compared to Δ*dinB*, expression of the *dinB(F13S)*, *dinB(Y79A)*, or *dinB(F13V)* alleles results in enhanced sensitivity to DinB cognate lesions regardless of whether they are expressed from a plasmid or from the chromosome (see Figs. 2, 3, and 4). Therefore, if survival upon MMS or NFZ treatment is TLS independent, *dinB(F13V)*, and especially *dinB(D103N)* should render ***Δ*** *dinB* strains sensitive to any DNA damaging agent regardless of the lesion it might bring about. UV damage was chosen to test this model because DinB is unable to bypass the thymine-thymine dimer lesions generated in the major grove of the DNA upon treatment [42]. 37 J/m^2^ of UV light, a dosage at which Δ*dinB* or *dinB^+^* strains are equally killed was used to treat Δ*dinB* strains with these *dinB* alleles. We find that ***Δ*** *dinB* cells bearing either the *dinB* catalytic or TLS deficient alleles survive as well as Δ*dinB* or *dinB^+^* strains upon UV treatment. In contrast, at comparable levels of MMS or NFZ treatment, Δ*dinB* strains expressing DinB(D103N) or DinB(F13V), show enhanced sensitivity to MMS or NFZ (Fig. 5). Thus, only DinB cognate lesions result in poor survival of Δ*dinB* cells expressing these *dinB* alleles, suggesting that survival in MMS or NFZ treatment is linked to TLS.

**Figure 6:**
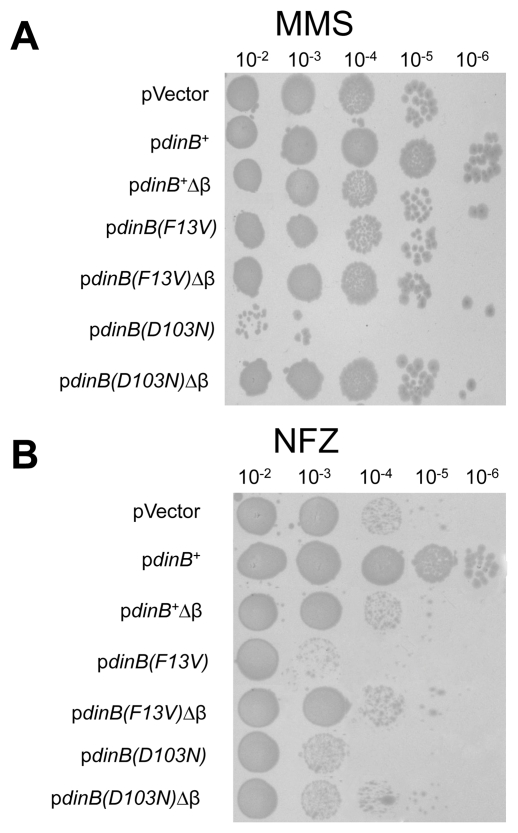
Δ*dinB* with variants lacking the residues comprising the β-clamp binding motif are no longer highly sensitive to MMS or NFZ. (**A**) A representative LB medium plate containing MMS (7.5 mM) with 10 fold serial dilutions of Δ*dinB* cells bearing the plasmid-borne *dinB* alleles is shown. (**B**) Same as (**A**) except cells were deposited on LB medium plates with NFZ (0.008 mM).

We next investigated whether the DinB variant-mediated enhanced sensitivity in MMS or NFZ requires the carboxy terminal residues known to interact with the processivity factor β-clamp [43]. Thus, derivatives of DinB(F13V), DinB(D103N), and DinB^+^, as a control, lacking the DinB β-clamp binding motif (^347^LVLGL^351^; [43], [44]) were constructed in the same low copy number plasmids (Table S1). If Δ*dinB* strains expressing DinB variants lacking the β-clamp binding motif are as sensitive to either MMS or NFZ as Δ*dinB*, then it could be inferred that the observed enhanced sensitivity is mediated through interactions with the β-clamp, and is consistent with the idea that these DinB variants are localized at the replication fork. We found that Δ*dinB* with p*dinB^+^*Δβ are more sensitive to MMS or NFZ compared to p*dinB* ^+^ (Fig. 6). In contrast, Δ*dinB* cells expressing DinB(D103N)Δβ on MMS or NFZ (Fig. 6) and those expressing DinB(F13V)Δβ on NFZ (Fig. 6B) are more resistant to these DNA damaging agents. These data suggest that the enhanced sensitivity observed with these *dinB* alleles is dependent on the β-clamp binding motif. This is perhaps the result of the interaction of DinB with the β-clamp, which is likely to be occurring at the replication fork.

From these independent sets of data we can deduce that the *in vivo* phenotypes observed in cells expressing *dinB* catalytic or other *dinB* alleles are the result of *in vivo* deficiencies in specific lesion bypass.

### DNA damage-induced mutation frequency as a measure of accurate TLS activity

In this report we present a DNA damage-induced mutagenesis screen with a substantial mutational target size [45], [46]. Cells are treated with either MMS or NFZ at concentrations where Δ*dinB* cells are equally killed, i.e. 7.5 mM MMS and 0.008 mM NFZ. Bacterial colonies that survive the treatment are then screened for loss of growth in minimal medium. A very conservative estimation of the target size is between 35 and 100 Kb, since any mutation that results in the inability to grow in minimal medium will be scored as a mutant. These include genes involved in amino acid, vitamin, or nucleotide biosynthesis. There is no selection in the detection of the mutant population that arises as the result of DNA damage and mutant colonies unable to grow in minimal medium are clonal.

**Figure 7:**
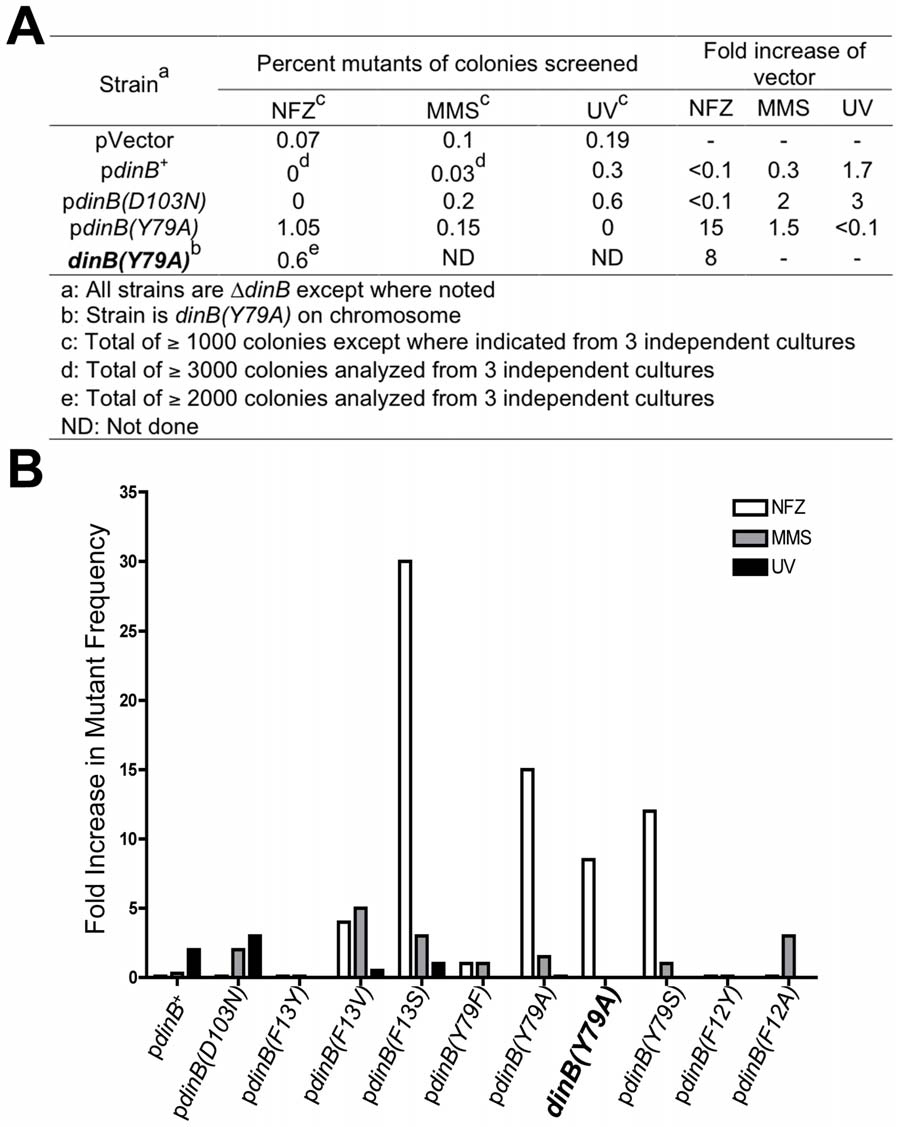
DNA damage-induced mutants. (**A**) Δ*dinB* with plasmid-borne DinB variants were treated with MMS (7.5 mM), NFZ (0.008 mM), or UV light (37 J/m^2^) and screened for mutants unable to grow on glucose minimal medium. Only Δ*dinB* strains carrying plasmid-borne *dinB^+^*, *dinB(D103N)*, *dinB(F13V)*, *dinB(F13S)*, or *dinB(Y79A)* alleles were assessed for UV-induced mutants. NFZ-induced mutants were also ascertained in the *dinB(Y79A*) strain in which the DinB variant is expressed from the chromosome (bold font). Mutants were equally distributed when independent cultures were analyzed. All samples have a standard error ≤5% of the average of mutants obtained per individual culture. (**B**) The fold difference shown in mutants is relative to Δ*dinB*.

We find that there is virtually no DNA damage-induced mutagenesis in Δ*dinB* expressing *dinB* ^+^ (Fig. 7). Importantly, although DinB is arguably at higher intracellular concentration than when expressed from a single chromosomal copy, it does not increase DNA damage-induced mutant frequency in this assay simply by being at a higher intracellular concentration. We also found low frequencies of mutants in Δ*dinB* and in Δ*dinB* expressing the catalytically inactive *dinB(D103N)* (Fig. 7), both of which are presumed to be the consequence of an activity independent from DinB.

Cells expressing *dinB* with mutations in the aromatic triad residues F13, Y79, and F12 display a frequency of MMS-induced mutants similar to Δ*dinB* or to the Δ*dinB*/p*dinB(D103N)* strain (Fig. 7). We find that Δ*dinB* cells expressing the DinB(F13V) variant have a modest increase in both MMS and NFZ-induced mutant frequency when compared to either Δ*dinB* or to those expressing DinB(D103N) (Fig. 7). Unexpectedly, there is a substantial increase in mutants for NFZ-treated Δ*dinB* strains expressing p*dinB(F13S)*, p*dinB(Y79A)*, or p*dinB(Y79S)* (Fig. 7). Notably, this increase is also observed in the *dinB(Y79A)* chromosomal strain (Fig. 7; bold font).

We also assessed the number of UV-induced mutants to validate the level of mutagenesis that is DinB-independent. We expected that UV-induced mutant frequencies would be similar to Δ*dinB* or Δ*dinB* strains expressing the catalytically inactive derivative DinB(D103N) after NFZ or MMS treatment. This is what was observed (Fig. 7) in the cases where it was determined. Intriguingly, we observed an increase in UV-induced mutants in strains expressing pDinB^+^ but not pDinB(D103N) (compare columns NFZ or MMS with UV for pDinB^+^ in Fig. 7A). This has been previously reported for DinB^+^, in an independent experiment in which selection for UV-induced Arg^+^ mutants was carried out [40]. Thus, there might be a role for DinB, or more likely DinB with its interacting partners [40], in regulating this mutagenesis.

### MMS induces the SOS-gene network more strongly than NFZ

The difference between NFZ- and MMS-induced mutant frequencies might be due to a fundamental distinction between the mechanisms regulating DinB alkylation or *N^2^*-dG lesion bypass. Perhaps there are other SOS induced proteins that might explain the elevated mutant frequency observed exclusively upon NFZ treatment. To measure the relative induction of the SOS-gene network in cells treated with MMS or NFZ, a green fluorescent protein (GFP; [47]) reporter plasmid was used. In this assay GFP is expressed under the regulation of the *sulA* gene promoter, an SOS-network gene [48], and GFP fluorescence is thus an indicator of SOS induction. This plasmid was introduced into both *dinB^+^* and Δ*dinB* strains by transformation. In this experiment ciprofloxacin [49] instead of UV irradiation was chosen as the SOS inducer to directly evaluate GFP fluorescence in a comparable time frame.

**Figure 8:**
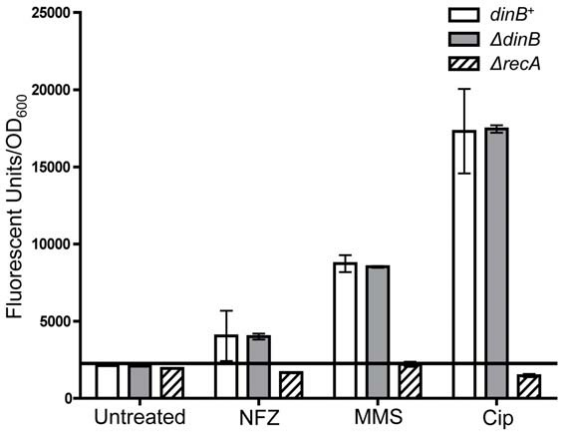
Relative induction of the SOS gene network in cells treated with MMS or NFZ. *dinB^+^*, Δ*dinB*, and Δ*recA* strains carrying a plasmid that expresses GFP from an SOS inducible promoter (*sulA*p-GFP) were treated with MMS (7.5 mM), NFZ (0.06 mM shown), or the strong SOS inducer ciprofloxacin (Cip) (0.1 µg/mL) [49]. *dinB^+^* and Δ*dinB* strains display a significant 50% increase, compared to the control Δ*recA* strain, in the ratio of Fluorescence/OD_600_ after 2 hours of NFZ treatment and after 1.5 hours of both MMS and ciprofloxacin treatments, 5 hours is shown. For 20 hour kinetic see Fig S2. No increased fluorescence is detected in NFZ concentrations below 0.06 mM (data not shown) when compared to untreated *dinB^+^* and Δ*dinB* cells, or the Δ*recA* negative control during the time frame the experiment was carried out (see Fig S2 for 20 hour kinetic). Error bars represent the standard deviation of the mean from at least 4 replicates.

We find that expression of GFP upon treatment with a DNA damaging agent is *dinB* independent (Figs. 8 and S2). Strikingly, we find that GFP fluorescence is greater in cells treated with MMS than in those treated with NFZ (Figs. 8 and S2). Moreover, no fluorescence was detected at NFZ concentrations lower than 0.06 mM, the concentration depicted in Fig. 8. Yet the NFZ concentration used to treat cells throughout this report is 7.5 times lower i.e. 0.008 mM, an NFZ concentration insufficient to induce the SOS response at levels similar to those measured in MMS treatment. Thus, it can be inferred that the large number of NFZ-induced mutants is specific to the *N^2^*-dG lesions and not due to overexpression of any other SOS-induced activity.

Taken together, this evidence indicates that the aromatic triad residues play different and nuanced roles in the TLS of MMS- and NFZ-induced lesions. The analyses carried out suggest that the active site is pliable and that the aromatic triad is essential for both bypass and accuracy.

## Discussion

Much has been learned about the Y family translesion (TLS) DNA polymerase DNA Pol IV (DinB) in *E. coli* [1], [6], [50]. This DNA polymerase inserts a nucleotide opposite specific DNA lesions (i.e. bypass or translesion synthesis activity) with relatively high accuracy compared to other Y DNA polymerases [15], [16]. Though under some conditions DinB has been shown to cause −1 frameshift mutations on misaligned templates [40], [51], [52], this appears to be regulated by protein-protein interactions [40], [51]. However, knowledge is lacking in regard to the role played by DinB's active site residues, in alkylation lesion bypass. Moreover, it is not known whether residues in the active site play a role in the accuracy of bypass of distinct DNA lesions. To fill this knowledge gap, we have undertaken structure/function analyses of the DinB active site and have gained insights into the active site residues that govern bypass and fidelity of different lesions. In the experiments reported here we use MMS and NFZ, reagents known to cause DNA lesions that kill cells without DinB [16], [19]. Specifically, we studied an aromatic residue triad F12, F13, and Y79 in the DinB active site (Fig. 1) by changing these conserved residues to ones of different size and polarity. Notably, this is the first report in which a number of these DinB variants have been studied when expressed from the chromosome.

The F13 residue is critical for both *in vivo* and *in vitro* DinB mediated bypass of *N^2^*-dG lesions [16]. Agreeing with previously published reports [16], expression of the DinB(F13V) variant in Δ*dinB* cells from a low copy number plasmid causes enhanced NFZ sensitivity (Fig. 2). This phenotypic signature is remarkably maintained when this *dinB* allele is expressed from the chromosome (Fig. 3). Notably, there is no enhanced sensitivity to MMS (Figs. 2 and 3), suggesting that the DinB active site adjusts to lesions. Thus, the relevance of different catalytic residues for bypass activity is likely to be lesion dependent.

We investigated whether changing the aromatic triad residues to different aromatic residues compromised the bypass activity of *E. coli* DinB. Our results show that the polarity of the aromatic residue is important for the F12 residue but not for the others (Fig. 4). It can be inferred that either a phenylalanine or a tyrosine at the F13 or Y79 positions allows for the insertion of a nucleotide opposite an *N^2^*-dG or an alkylation lesion, resulting in no change in the activity of the DNA polymerase. A comparable result has been found in B-family DNA polymerases [53], [54] when a similarly positioned residue was changed from a tyrosine to a phenylalanine. Based on the analysis carried out in Dpo4 [55], it is probable that the DinB(F12Y) mutation reduces hydrophobic packing, which in turn leads to reduced TLS; however, in the Y79F mutation, the phenylalanine is able to stabilize the F13 residue allowing for efficient TLS. A recent study demonstrated that the active site residue Y112 (F13 in DinB) of the human DinB counterpart, Pol κ, is required not only for effective bypass of certain lesions and exclusion of rNTPs from DNA synthesis, but also for mismatch-primer extension [56]. It is plausible that mutations in either F111 (F12) or Y174 (Y79) could abolish the ability of Pol κ to carry out any of these functions.

We further analyzed these three key aromatic residues by assaying survival of Δ*dinB* strains bearing plasmid-borne DinB variants in which each of the aromatic triad residues were mutated to amino acids without aromatic rings. None of these variants rescued *ΔdinB* strains upon MMS or NFZ treatment, a phenotype that was maintained independently of the allele location (Figs. 3 and 4), suggesting that all three aromatic residues are essential for bypass of both alkylation lesions and *N^2^*-dG minor groove adducts.

It was plausible that poor survival in NFZ or MMS by Δ*dinB* cells expressing the various *dinB* deficient alleles might be due to increased intracellular DinB concentrations [57] and not necessarily to TLS. If the variants were causing lethality due to, for example, unregulated access to stalled replication forks; it should be observed independent of both the treatment and the *dinB* deficient allele tested. However, this is not the case (Fig. 5). Provocatively, the poor survival phenotype is only observed when cells are treated with either NFZ or MMS, but not with UV (Fig. 5). We (Fig. 2), and others [16], [19] have shown that DinB is necessary for survival in alkylation or *N^2^*-dG DNA damage, demonstrating that MMS- and NFZ-induced lesions are cognate DinB lesions, i.e. DinB activity is critical for survival. The same is true for other DinB-like polymerases [20], [21]. Differences in survival are only observed when specific cognate lesions are present on the DNA, suggesting that lesions might actively recruit DinB polymerases to the replication fork, possibly increasing the local polymerase concentration. The localized concentration of DinB would then permit efficient exchange with the replicative polymerase, probably via the β-clamp [58]. This suggests that lesion specificity might play an important role in the TLS activity of DinB. This concept of lesion-induced recruitment of Y family DNA polymerases is similar to that occurring during somatic hypermutation ([59] and references therein).

Importantly, the SOS gene network is robustly induced with MMS but not so with NFZ. Indeed, at least a 7.5 fold higher NFZ concentration than the one used here to routinely treat cells with was required to detect any SOS induction (Fig. 8). Interestingly, nitrofurantoin, another reagent within the class of activated nitrofurans [60] is also a poor inducer of the SOS response [61], [62], [63]. Thus, intracellular concentrations of DinB and other SOS-induced proteins would be higher in MMS than in NFZ treated cells. These results suggest that it is DinB and not other SOS induced proteins that are responsible for the observed loss in survival of Δ*dinB* cells expressing *dinB* catalytic or TLS deficient alleles (Figs. 2, 3, and 4). Moreover, the phenotypic signature of some of these deficient alleles is maintained when crossed onto the chromosome (Fig. 3) suggesting that it is not exclusively due to high intracellular concentrations. Finally, the DinB protein appears to be properly localized at or near the replication fork since cells carrying the *dinB(F13V)*Δβ or *dinB(D103N)*Δβ alleles, which lack the conserved motif that permits DinB-β clamp interactions, are no longer highly sensitive to MMS or NFZ (Fig. 6). From these data we can infer that DinB and its variants have to be at or near the replication fork to effect either survival or lethality of cells upon treatment with reagents producing DinB cognate lesions.

The analysis we have carried out indicates the aromatic triad in the active site of DinB is needed for cells to survive NFZ or MMS treatment because mutations in the aromatic triad impair *in vivo* TLS.

Additionally, we report here that the aromatic triad is important for the accuracy of DinB bypass. The MMS- or UV-induced mutant frequency is quite low for Δ*dinB* strains carrying any of the plasmid-borne DinB variants. Conversely, an NFZ-induced mutator phenotype is apparent in Δ*dinB* cells carrying the DinB(Y79A), DinB(Y79S), or DinB(F13S) derivatives. A similar result is obtained with strains in which DinB(Y79A) is expressed from the chromosome (Fig. 7). There are two possibilities as to why only NFZ induced mutagenesis is observed: (1) low fidelity on undamaged DNA, as some of these variants are known to be incapable of TLS *in vitro* [15], [16], and/or (2) mutagenesis is the product of the *in vivo* TLS activity of these DinB variants. It is possible the *in vitro* and *in vivo* properties observed in DinB(Y79) variants are due to the inability of the new residue at position 79 to properly support F13 in carrying out high fidelity bypass. Although the substitution of valine for phenylalanine at position 13 does render DinB TLS deficient, it is only when a smaller serine is substituted for phenylalanine that the fidelity of the polymerase is severely compromised. All of these changes in either the F13 or Y79 do not affect the bypass fidelity in MMS, presumably because the active site is flexible and adapts to the different lesions. Perhaps the fidelity of alkylation lesion bypass is regulated differently from the fidelity of NFZ-induced lesion bypass.

The observed NFZ-induced mutants are also likely DinB dependent. If the mutants were occurring as a result of the action of another polymerase, i.e. DNA Pol V, it would have been evident in the UV-induced mutagenesis assay. UV treated cells expressing DinB variants, display a number of DNA damage-induced mutants that are, for the most part, equivalent to the level of mutants found both in Δ*dinB* carrying the DinB(D103N) variant, which is unable to synthesize DNA, and to cells without DinB (Fig. 7). Furthermore, there is evidence that DNA Pol II is not involved in the bypass of MMS- or NFZ-derived lesions in cells that are proficient for base or nucleotide excision repair [19], [64], making it difficult to envision a simple model in which DNA Pol II is responsible for the observed DNA damage-induced mutagenesis. We carried out Illumina deep sequencing of the genome of several independent MMS treated Δ*dinB/*p*dinB(D103N)* or *pdinB(F13V)* strains that we identified as mutants based on their inability to grow in minimal medium. Strikingly, MMS-induced mutants of Δ*dinB*/p*dinB(D103N)* have only single base pair substitutions (SNPs), including those in genes which could be responsible for the lack of growth in minimal medium (data not shown). Notably, both SNPs and −1 frameshifts (the mutational signature of DinB(F13V) [40]) were detected in mutants derived from Δ*dinB/*p*dinB(F13V)* strains. This evidence further suggests that DNA Pol IV and its variants are responsible for effecting mutagenesis.

There is growing evidence for a role of DinB-like polymerases in human cancers [65], [66], [67], [68]. Thus, this triad of aromatic residues in the DinB active site might be playing similar roles in DinB homologues especially regarding fidelity. When compared to the *in silico* model of DinB, the Pol κ crystal structure shows that the aromatic triad is identical in conformation (DinB F12, F13 and Y79 are homologous to Pol κ F111, Y112, and Y174; Fig. 1). Notably, in the 1000 Genomes database [69] we find that there is only one known polymorphism in the protein sequence of Pol κ that is homologous to *E. coli* DinB, (S423R), which is not an active site residue. The lack of variations in the Pol κ sequences, especially in the active site, agrees with data showing natural populations of *E. coli* select against polymorphisms in the DinB catalytic domain [70]. The mutator phenotypes observed in cells expressing DinB(F13S, Y79S, or Y79A) also indicate that variations in homologous residues of Pol κ could lead to a similar reduction in TLS fidelity. The lack of polymorphisms in humans might also be due to selection against such changes, perhaps the result of embryonic lethality.

Thus, the analyses of the DinB active site and its aromatic triad have provided insights into mechanisms that govern both TLS and the fidelity of the bypass of different cognate lesions. In this regard, we found a strikingly low level of DNA damage-induced mutants in Δ*dinB* cells expressing wild type DinB from a low copy number plasmid, despite it both being at a higher copy number than chromosomal, and having a sizable mutational target. Furthermore, we found few alkylation DNA damage-induced mutants, in agreement with previous findings [19]. Finally, we found that the aromatic triad plays a key role in the bypass fidelity of NFZ-induced lesions. This supports the notion that *N^2^*-dG lesions might indeed be the preferred lesions recognized and bypassed by this DNA polymerase. Although bacteria may encounter NFZ as an antibiotic in the treatment of infections [71], we are still left with the question: what is the endogenous source of *N^2^*-dG lesions? We have no direct answer yet to this question, however, since DinB-like DNA polymerases are evolutionarily conserved, the source of their preferred *N^2^*-dG lesion substrate must be the result of an ordinary metabolite. One exciting candidate is methylglyoxal, a byproduct of glycolysis that can form *N* ^2^-(1-carboxyethyl)-2′-deoxyguanosine (*N^2^*-CEdG) lesions that are bypassed by DinB and human Pol κ [72].

We have shown here that the high fidelity of DinB is apparent upon alkylation damage, an inescapable and pervasive form of DNA damage, even when the DinB active site performs *in vivo* error-prone NFZ-induced lesion bypass. In addition, we propose that it is the nature of the lesion that localizes DinB to the replication fork and facilitates protein-protein interactions to prompt DNA polymerase exchange with the replicative DNA polymerase when it has stalled.

## Materials and Methods

### Bacterial Strains and Plasmids

Bacterial strains and plasmids used in this report are listed in Table S1. The P90C Δ*dinB* strain was generated by P1 transduction using an allele from the KEIO collection [73] (a kind gift of the Lewis lab at NEU). Plasmid-borne DinB mutants were constructed using the GeneTailor Site-Directed Mutagenesis System (Invitrogen) and introduced into CaCl_2_ chemically competent cells by transformation [74]. Mutagenic oligonucleotides are listed in Table S1. Mutations were verified by DNA sequencing, carried out at the Tufts University Core Facility in Boston, MA.

### Survival Assays

Cultures were grown to saturation in either liquid LB or M9 minimal medium [74] with ampicillin (Amp, 200 µg/mL; Sigma). Serial dilutions of saturated cultures were treated with varying concentrations of methyl methanesulfonate (MMS, 5, 7.5, and 10 mM; Acros Organics), nitrofurazone (NFZ, 0.008 mM; Sigma), or were irradiated in minimal medium at a UV (254 nm) intensity of 37 J/m^2^.

### Construction of DinB Variants in the Chromosome

*dinB(D103N)*, *dinB(F13V)*, *dinB(F13S)*, and *dinB(Y79A)* alleles were introduced into the chromosome using the SOE-LRed method [75].

### Mutation Assays

Cells were evenly spread with glass beads onto LB medium with 7.5 mM MMS or 0.008 mM NFZ. These concentrations of MMS and NFZ equally killed Δ*dinB* cells. UV irradiation (∼37 J/m^2^) was carried out on M9 minimal medium supplemented with casaminoacids [45]. Under these conditions, this level of irradiation killed Δ*dinB* and *dinB* ^+^ cells to the same extent. Surviving colonies were screened for loss of function on minimal medium without amino acid supplementation [45], except for proline [76], which is required by the parental strain. Three independent experiments were carried out per DinB derivative until a minimum of 1000 colonies were screened.

### SOS Induction Assays

Cells bearing a plasmid expressing GFP from a *sulA* promoter (pUA66-*sulA*, [47]) were grown to saturation in minimal medium with Kanamycin (35 µg/mL; Sigma). These cultures were diluted 1 to 10 in the same growth medium with the appropriate concentration of MMS (7.5 mM), NFZ (0.008–0.06 mM), or ciprofloxacin (0.1 µg/mL, Cip; Sigma) in 96 well black plates with clear flat bottom (Corning). The plates were incubated at 37°C with intermittent shaking for 20 hours. GFP fluorescence (485/528 nm; Excitation/Emission) and turbidity (600 nm) were measured every 5 minutes with in a BioTek Synergy HT-I plate reader.

## Supporting Information

Figure S1 **Kinetic of MMS lethality of Δ** ***dinB*** **strains harboring either** ***dinB*** **^**+**^ or catalytic/translesion deficient** ***dinB*** **alleles.** Only the plasmid-borne *dinB* ^+^ allele rescues Δ*dinB* MMS sensitivity. Neither plasmid-borne DinB(D103N) nor DinB(F13V) rescue Δ*dinB* cells treated with various concentrations of MMS. Enhanced sensitivity is observed in Δ*dinB* strains expressing DinB(D103N) when compared to Δ*dinB*. Error bars represent the standard deviation of the mean from at least 3 independent experiments.
(TIF)

Figure S2 **MMS is a more robust inducer of the SOS response than NFZ.** Kinetic of the ratio of fluorescence over OD_600_ is shown for *dinB^+^*, Δ*dinB*, and Δ*recA* strains carrying p*sulA*p-GFP. Strains were treated with MMS (7.5 mM), NFZ (0.06 mM shown), or Cip (0.1 µg/mL). Fluorescence readings and optical density (600 nM) were taken every 5 minutes for 20 hours in a plate reader. Data shown are the average of at least 4 replicates, and the standard deviation of the mean is ≤25% for all samples.
(TIF)

Table S1 **Strain, Plasmid, and Oligonucleotide Table.**
(TIF)

## References

[1] Friedberg EC, Walker GC, Siede W, Wood RD, Schultz RA (2006). DNA repair and mutagenesis.

[2] Friedberg EC, Fischhaber PL, Kisker C (2001). Error-prone DNA polymerases: novel structures and the benefits of
infidelity.. Cell.

[3] Sutton MD, Smith BT, Godoy VG, Walker GC (2000). The SOS response: recent insights into
*umuDC*-dependent mutagenesis and DNA damage
tolerance.. Annu Rev Genet.

[4] Bonner CA, Hays S, McEntee K, Goodman MF (1990). DNA polymerase II is encoded by the DNA damage-inducible
*dinA* gene of *Escherichia
coli*.. Proc Natl Acad Sci U S A.

[5] Napolitano R, Janel-Bintz R, Wagner J, Fuchs RP (2000). All three SOS-inducible DNA polymerases (Pol II, Pol IV and Pol
V) are involved in induced mutagenesis.. Embo J.

[6] Ohmori H, Friedberg EC, Fuchs RP, Goodman MF, Hanaoka F (2001). The Y-family of DNA polymerases.. Mol Cell.

[7] Rattray AJ, Strathern JN (2003). Error-prone DNA polymerases: when making a mistake is the only
way to get ahead.. Annu Rev Genet.

[8] Fuchs RP, Fujii S, Wagner J (2004). Properties and functions of *Escherichia coli*:
Pol IV and Pol V.. Adv Protein Chem.

[9] Nohmi T (2006). Environmental stress and lesion-bypass DNA
polymerases.. Annu Rev Microbiol.

[10] Kim SR, Matsui K, Yamada M, Gruz P, Nohmi T (2001). Roles of chromosomal and episomal *dinB* genes
encoding DNA pol IV in targeted and untargeted mutagenesis in
*Escherichia coli*.. Mol Genet Genomics.

[11] Kwon-Shin O, Bodner JB, McHenry CS, Bambara RA (1987). Properties of initiation complexes formed between
*Escherichia coli* DNA polymerase III holoenzyme and
primed DNA in the absence of ATP.. J Biol Chem.

[12] Shavitt O, Livneh Z (1989). The beta subunit modulates bypass and termination at UV lesions
during *in vitro* replication with DNA polymerase III
holoenzyme of *Escherichia coli*.. J Biol Chem.

[13] Bunting KA, Roe SM, Pearl LH (2003). Structural basis for recruitment of translesion DNA polymerase
Pol IV/DinB to the beta-clamp.. Embo J.

[14] Wagner J, Fujii S, Gruz P, Nohmi T, Fuchs RP (2000). The beta clamp targets DNA polymerase IV to DNA and strongly
increases its processivity.. EMBO Rep.

[15] Jarosz DF, Cohen SE, Delaney JC, Essigmann JM, Walker GC (2009). A DinB variant reveals diverse physiological consequences of
incomplete TLS extension by a Y-family DNA polymerase.. Proc Natl Acad Sci U S A.

[16] Jarosz DF, Godoy VG, Delaney JC, Essigmann JM, Walker GC (2006). A single amino acid governs enhanced activity of DinB DNA
polymerases on damaged templates.. Nature.

[17] Panigrahi GB, Walker IG (1990). The N^2^-guanine adduct but not the
C^8^-guanine or N^6^-adenine adducts formed by
4-nitroquinoline 1-oxide blocks the 3′-5′ exonuclease action of
T4 DNA polymerase.. Biochemistry.

[18] Whiteway J, Koziarz P, Veall J, Sandhu N, Kumar P (1998). Oxygen-insensitive nitroreductases: analysis of the roles of
*nfsA* and *nfsB* in development of
resistance to 5-nitrofuran derivatives in *Escherichia
coli*.. J Bacteriol.

[19] Bjedov I, Dasgupta CN, Slade D, Le Blastier S, Selva M (2007). Involvement of *Escherichia coli* DNA polymerase
IV in tolerance of cytotoxic alkylating DNA lesions *in
vivo*.. Genetics.

[20] Johnson RE, Yu SL, Prakash S, Prakash L (2007). A role for yeast and human translesion synthesis DNA polymerases
in promoting replication through 3-methyl adenine.. Mol Cell Biol.

[21] Plosky BS, Frank EG, Berry DA, Vennall GP, McDonald JP (2008). Eukaryotic Y-family polymerases bypass a
3-methyl-2′-deoxyadenosine analog *in vitro* and methyl
methanesulfonate-induced DNA damage *in
vivo*.. Nucleic Acids Res.

[22] Kyrtopoulos SA (1998). DNA adducts in humans after exposure to methylating
agents.. Mutat Res.

[23] Lawley PD (1966). Effects of some chemical mutagens and carcinogens on nucleic
acids.. Prog Nucleic Acid Res Mol Biol.

[24] Lindahl T (1993). Instability and decay of the primary structure of
DNA.. Nature.

[25] Singer B, Kusmierek JT (1982). Chemical mutagenesis.. Annu Rev Biochem.

[26] Lengeler J (1980). Analysis of the physiological effects of the antibiotic
streptozotocin on *Escherichia coli* K 12 and other sensitive
bacteria.. Arch Microbiol.

[27] Murray-Lyon IM, Eddleston AL, Williams R, Brown M, Hogbin BM (1968). Treatment of multiple-hormone-producing malignant islet-cell
tumour with streptozotocin.. Lancet.

[28] Grzesiuk E, Gozdek A, Tudek B (2001). Contribution of *E. coli* AlkA, TagA glycosylases
and UvrABC-excinuclease in MMS mutagenesis.. Mutat Res.

[29] Lindahl T, Wood RD (1999). Quality control by DNA repair.. Science.

[30] Monti P, Iannone R, Campomenosi P, Ciribilli Y, Varadarajan S (2004). Nucleotide excision repair defect influences lethality and
mutagenicity induced by Me-lex, a sequence-selective N^3^-adenine
methylating agent in the absence of base excision repair.. Biochemistry.

[31] Rebeck GW, Samson L (1991). Increased spontaneous mutation and alkylation sensitivity of
*Escherichia coli* strains lacking the
*ogt* O^6^-methylguanine DNA repair
methyltransferase.. J Bacteriol.

[32] Sedgwick B (2004). Repairing DNA-methylation damage.. Nat Rev Mol Cell Biol.

[33] Kim HS, LeBreton PR (1994). UV photoelectron and ab initio quantum mechanical
characterization of valence electrons in
Na(+)-water-2′-deoxyguanosine 5′-phosphate clusters:
electronic influences on DNA alkylation by methylating and ethylating
carcinogens.. Proc Natl Acad Sci U S A.

[34] Wyatt MD, Pittman DL (2006). Methylating agents and DNA repair responses: Methylated bases and
sources of strand breaks.. Chem Res Toxicol.

[35] Larson K, Sahm J, Shenkar R, Strauss B (1985). Methylation-induced blocks to *in vitro* DNA
replication.. Mutat Res.

[36] Shah D, Kelly J, Zhang Y, Dande P, Martinez J (2001). Evidence in *Escherichia coli* that
N^3^-methyladenine lesions induced by a minor groove binding methyl
sulfonate ester can be processed by both base and nucleotide excision
repair.. Biochemistry.

[37] Singer B, Brent TP (1981). Human lymphoblasts contain DNA glycosylase activity excising N-3
and N-7 methyl and ethyl purines but not O^6^-alkylguanines or
1-alkyladenines.. Proc Natl Acad Sci U S A.

[38] Vasquez-Del Carpio R, Silverstein TD, Lone S, Swan MK, Choudhury JR (2009). Structure of human DNA polymerase kappa inserting dATP opposite
an 8-OxoG DNA lesion.. PLoS ONE.

[39] Silverstein TD, Johnson RE, Jain R, Prakash L, Prakash S Structural basis for the suppression of skin cancers by DNA
polymerase eta.. Nature.

[40] Godoy VG, Jarosz DF, Simon SM, Abyzov A, Ilyin V (2007). UmuD and RecA directly modulate the mutagenic potential of the Y
family DNA polymerase DinB.. Mol Cell.

[41] Wagner J, Gruz P, Kim SR, Yamada M, Matsui K (1999). The *dinB* gene encodes a novel *E.
coli* DNA polymerase, DNA pol IV, involved in
mutagenesis.. Mol Cell.

[42] Tang M, Pham P, Shen X, Taylor JS, O'Donnell M (2000). Roles of *E. coli* DNA polymerases IV and V in
lesion-targeted and untargeted SOS mutagenesis.. Nature.

[43] Dalrymple BP, Kongsuwan K, Wijffels G, Dixon NE, Jennings PA (2001). A universal protein-protein interaction motif in the eubacterial
DNA replication and repair systems.. Proc Natl Acad Sci U S A.

[44] Becherel OJ, Fuchs RP, Wagner J (2002). Pivotal role of the beta-clamp in translesion DNA synthesis and
mutagenesis in *E. coli* cells.. DNA Repair (Amst).

[45] Godoy VG, Gizatullin FS, Fox MS (2000). Some features of the mutability of bacteria during nonlethal
selection.. Genetics.

[46] Neidhardt FC (1996). *Escherichia coli* and *Salmonella* :
cellular and molecular biology.

[47] Zaslaver A, Bren A, Ronen M, Itzkovitz S, Kikoin I (2006). A comprehensive library of fluorescent transcriptional reporters
for *Escherichia coli*.. Nat Methods.

[48] Godoy VG, Beuning PJ, Walker GC, Lennarz WJaL, M. D. (2005). Gene Expression in Bacterial Systems: The LexA Regulatory
System.. Encyclopedia of Biological Chemistry.

[49] Dorr T, Lewis K, Vulic M (2009). SOS response induces persistence to fluoroquinolones in
*Escherichia coli*.. PLoS Genet.

[50] Andersson DI, Koskiniemi S, Hughes D (2010). Biological roles of translesion synthesis DNA polymerases in
eubacteria.. Mol Microbiol.

[51] Foti JJ, Delucia AM, Joyce CM, Walker GC (2010). UmuD(2) inhibits a non-covalent step during DinB-mediated
template slippage on homopolymeric nucleotide runs.. J Biol Chem.

[52] Rosenberg SM, Longerich S, Gee P, Harris RS (1994). Adaptive mutation by deletions in small mononucleotide
repeats.. Science.

[53] Bonnin A, Lazaro JM, Blanco L, Salas M (1999). A single tyrosine prevents insertion of ribonucleotides in the
eukaryotic-type phi29 DNA polymerase.. J Mol Biol.

[54] Yang G, Franklin M, Li J, Lin TC, Konigsberg W (2002). A conserved Tyr residue is required for sugar selectivity in a
Pol alpha DNA polymerase.. Biochemistry.

[55] DeLucia AM, Grindley ND, Joyce CM (2003). An error-prone family Y DNA polymerase (DinB homolog from
*Sulfolobus solfataricus*) uses a ‘steric
gate’ residue for discrimination against
ribonucleotides.. Nucleic Acids Res.

[56] Niimi N, Sassa A, Katafuchi A, Gruz P, Fujimoto H (2009). The steric gate amino acid tyrosine 112 is required for efficient
mismatched-primer extension by human DNA polymerase kappa.. Biochemistry.

[57] Uchida K, Furukohri A, Shinozaki Y, Mori T, Ogawara D (2008). Overproduction of *Escherichia coli* DNA
polymerase DinB (Pol IV) inhibits replication fork progression and is
lethal.. Mol Microbiol.

[58] Sutton MD, Duzen JM (2006). Specific amino acid residues in the beta sliding clamp establish
a DNA polymerase usage hierarchy in *Escherichia
coli*.. DNA Repair (Amst).

[59] Burch LH, Yang Y, Sterling JF, Roberts SA, Chao FG (2011). Damage-induced localized hypermutability.. Cell Cycle.

[60] Tu Y, McCalla DR (1975). Effect of activated nitrofurans on DNA.. Biochim Biophys Acta.

[61] Sengupta S, Rahman MS, Mukherjee U, Basak J, Pal AK (1990). DNA damage and prophage induction and toxicity of nitrofurantoin
in *Escherichia coli* and *Vibrio cholerae*
cells.. Mutat Res.

[62] Bi E, Lutkenhaus J (1990). Analysis of *ftsZ* mutations that confer
resistance to the cell division inhibitor SulA (SfiA).. J Bacteriol.

[63] Maguin E, Lutkenhaus J, D'Ari R (1986). Reversibility of SOS-associated division inhibition in
*Escherichia coli*.. J Bacteriol.

[64] Ona KR, Courcelle CT, Courcelle J (2009). Nucleotide excision repair is a predominant mechanism for
processing nitrofurazone-induced DNA damage in *Escherichia
coli*.. J Bacteriol.

[65] Betous R, Rey L, Wang G, Pillaire MJ, Puget N (2009). Role of TLS DNA polymerases eta and kappa in processing naturally
occurring structured DNA in human cells.. Mol Carcinog.

[66] Pillaire MJ, Selves J, Gordien K, Gourraud PA, Gentil C (2010). A ‘DNA replication’ signature of progression and
negative outcome in colorectal cancer.. Oncogene.

[67] Stallons LJ, McGregor WG (2010). Translesion Synthesis Polymerases in the Prevention and Promotion
of Carcinogenesis.. J Nucleic Acids.

[68] Wang H, Wu W, Wang HW, Wang S, Chen Y (2010). Analysis of specialized DNA polymerases expression in human
gliomas: association with prognostic significance.. Neuro Oncol.

[69] Durbin RM, Abecasis GR, Altshuler DL, Auton A, Brooks LD (2010). A map of human genome variation from population-scale
sequencing.. Nature.

[70] Bjedov I, Lecointre G, Tenaillon O, Vaury C, Radman M (2003). Polymorphism of genes encoding SOS polymerases in natural
populations of *Escherichia coli*.. DNA Repair (Amst).

[71] Rodgers GL, Mortensen JE, Fisher MC, Long SS (1997). *In vitro* susceptibility testing of topical
antimicrobial agents used in pediatric burn patients: comparison of two
methods.. J Burn Care Rehabil.

[72] Yuan B, Cao H, Jiang Y, Hong H, Wang Y (2008). Efficient and accurate bypass of
N^2^-(1-carboxyethyl)-2′-deoxyguanosine by DinB DNA
polymerase *in vitro* and *in
vivo*.. Proc Natl Acad Sci U S A.

[73] Baba T, Ara T, Hasegawa M, Takai Y, Okumura Y (2006). Construction of *Escherichia coli* K-12 in-frame,
single-gene knockout mutants: the Keio collection.. Mol Syst Biol.

[74] Miller JH (1972). Experiments in molecular genetics.

[75] Benson RW, Cafarelli TM, Godoy VG (2011). SOE-LRed: A simple and time-efficient method to localize genes
with point mutations onto the *Escherichia coli*
chromosome.. J Microbiol Methods.

[76] Miller JH (1992). A short course in bacterial genetics : a laboratory manual and handbook
for *Escherichia coli* and related bacteria.

