# Supplementary figures and images for: An Active Site Aromatic Triad in Escherichia coli DNA Pol IV Coordinates Cell Survival and Mutagenesis in Different DNA Damaging Agents
Source: PLoS One. 2011 May 17;6(5):e19944. doi: 10.1371/journal.pone.0019944 (PMC3096655; doi:10.1371/journal.pone.0019944)

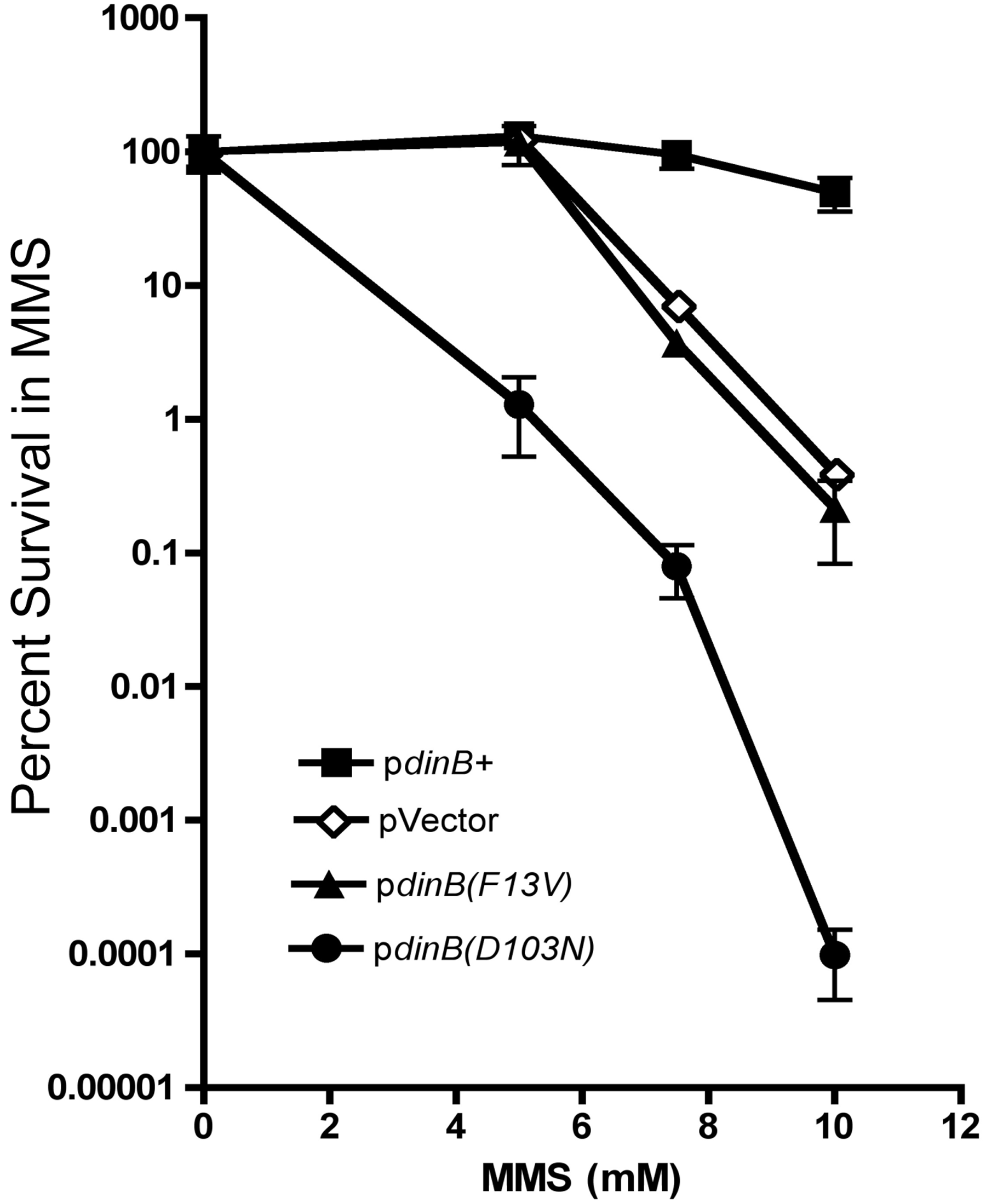

Supplement: Figure S1 — Kinetic of MMS lethality of Δ dinB strains harboring either dinB + or catalytic/translesion deficient dinB alleles. Only the plasmid-borne dinB + allele rescues ΔdinB MMS sensitivity. Neither plasmid-borne DinB(D103N) nor DinB(F13V) rescue ΔdinB cells treated with various concentrations of MMS. Enhanced sensitivity is observed in ΔdinB strains expressing DinB(D103N) when compared to ΔdinB. Error bars represent the standard deviation of the mean from at least 3 independent experiments. (TIF) [file pone.0019944.s001.tif]

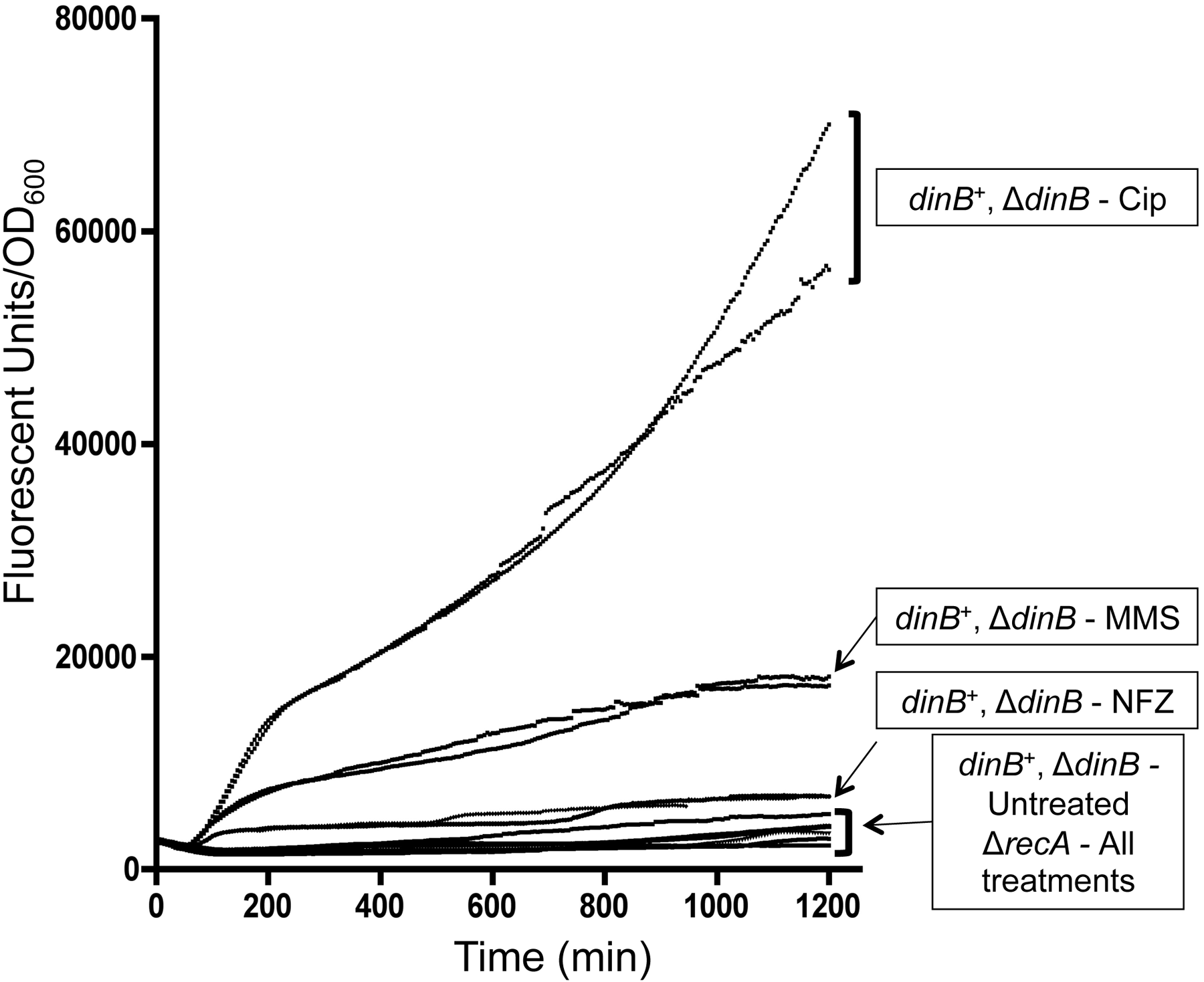

Supplement: Figure S2 — MMS is a more robust inducer of the SOS response than NFZ. Kinetic of the ratio of fluorescence over OD600 is shown for dinB+, ΔdinB, and ΔrecA strains carrying psulAp-GFP. Strains were treated with MMS (7.5 mM), NFZ (0.06 mM shown), or Cip (0.1 µg/mL). Fluorescence readings and optical density (600 nM) were taken every 5 minutes for 20 hours in a plate reader. Data shown are the average of at least 4 replicates, and the standard deviation of the mean is ≤25% for all samples. (TIF) [file pone.0019944.s002.tif]
